# Review and classification of variability analysis techniques with clinical applications

**DOI:** 10.1186/1475-925X-10-90

**Published:** 2011-10-10

**Authors:** Andrea Bravi, André Longtin, Andrew JE Seely

**Affiliations:** 1 Department of Cellular and Molecular Medicine, University of Ottawa, Ottawa, Ontario, Canada; 2 Department of Physics, University of Ottawa, Ottawa, Ontario, Canada; 3 Division of Thoracic Surgery, University of Ottawa, Ottawa, Ontario, Canada; 4 Department of Critical Care Medicine, University of Ottawa, Ottawa, Ontario, Canada

## Abstract

Analysis of patterns of variation of time-series, termed variability analysis, represents a rapidly evolving discipline with increasing applications in different fields of science. In medicine and in particular critical care, efforts have focussed on evaluating the clinical utility of variability. However, the growth and complexity of techniques applicable to this field have made interpretation and understanding of variability more challenging. Our objective is to provide an updated review of variability analysis techniques suitable for clinical applications. We review more than 70 variability techniques, providing for each technique a brief description of the underlying theory and assumptions, together with a summary of clinical applications. We propose a revised classification for the domains of variability techniques, which include statistical, geometric, energetic, informational, and invariant. We discuss the process of calculation, often necessitating a mathematical transform of the time-series. Our aims are to summarize a broad literature, promote a shared vocabulary that would improve the exchange of ideas, and the analyses of the results between different studies. We conclude with challenges for the evolving science of variability analysis.

## Introduction

Variability analysis can be defined as the comprehensive assessment of the degree and character of patterns of variation over time intervals. This analysis found applications in many different research fields, from weather forecasting [1], to network analysis [2], process monitoring [3] and medicine, the subject of this paper. Considering the systemic host response to trauma, shock, or sepsis as a complex system, Seely et al. broadly hypothesized that the analysis of multiorgan variability (patterns of variation over time) and connectivity (patterns of interconnection over space) of physiological signals offer a means to track the system state of the host response, offering the potential for early detection of deterioration and improved real-time prognostication [3,4]. Rooted in nonlinear dynamics and physics, the approach of variability analysis has been successfully applied also for the prediction of mortality after acute myocardial infarction [5,6], detection of sleep apnea [7,8], assessment of the autonomic nervous system activity [9,10] and evaluation of the circadian rhythms regulating the body [11,12]. In particular, there is an increasing interest in the application of variability monitoring to improve clinical outcomes [13].

There has been extensive research to develop multiple measures of variability and trying to characterize how variability changes with respect to specific stimuli; however, our objective in this paper is to take a step back and assess the array of variability techniques available today, analyze their properties and clinical applications, and re-evaluate their classification.

The most specific classification sees several domains of variability such as the time domain, the frequency domain, the entropy domain and the scale-invariant domain [13]. However, in many recent papers the classification is often reduced to only time domain, frequency domain and a more general nonlinear domain [9,14-17]. Therefore, despite the several years passed, the classification of variability techniques has not significantly evolved since the important work of the Task Force for heart rate variability [18]. The Task Force itself was created to provide guidelines that were out of date. For instance, they recommended the equipment designed to analyze heart rate variability to include three specific statistical measures, plus one specific geometrical measure. A new classification system is therefore needed to create new guidelines; one that is capable of giving a place to the increased number of techniques that currently are not classified.

The objective of the present paper is to present the variability techniques currently available in the biomedical domain, with an emphasis on the ones used for the analysis of physiological signals in clinical settings. Furthermore, a new classification of these techniques is proposed with the aim of categorizing the knowledge collected in different fields using variability, giving the possibility to new researchers approaching variability analysis to orient themselves in the analysis of their results, and aiding the exchange of ideas. The paper starts with the explanation of the proposed classification, useful for the comprehension of the variability techniques. Then, each technique is presented, highlighting its assumptions, giving an intuitive explanation of how to compute it, along with examples of application in clinical contexts. The majority of applications were found related to the study of cardiac diseases. The reason is that, even if the research for our review did not specifically target the cardiac realm, the heart rate is the most commonly studied biomedical time series using the techniques described here, due to the ease of obtaining the data and their relationship with autonomic activity [18]. The paper concludes with the presentation of two major challenges in variability analysis and the conclusions. In the following, "time series" is meant as a set of measurements of one variable. When the context is the simultaneous measurement of multiple variables, we will use the term multivariate time series.

## Classification

After the work of the Task Force of 1996 [18], there has been considerable development of novel techniques for variability analysis. As physiologic time-series are remarkably complex, authors have sought to distill the information into a single measure or graphical representation that uncovers the underlying complexity. To begin our analysis we highlight two main categories of variability analysis techniques: *Transformations *and *Features*. Those techniques share in common the potential to manipulate data in order to identify certain properties of interest. Starting from the former, we here define a transformation as mathematicians would do, i.e. as a function which maps the samples of dataset from one set of values to another. Examples of transformation techniques are Recurrence plots [19,20], Poincaré plots [9] and Symbolic Dynamics [21]; others will be shown below. The principle underlying transformations is to rearrange data in ways allowing the extraction of additional features, otherwise difficult to detect. The payoff is that the enhancement of a certain type of information weakens the detection of other types of information.

Several transformations have been devised by a range of scientists for time series analysis; we categorized them in two different sets due to their conceptual difference. The first one is what we call the quantitative transformations. All the techniques belonging to this set make use of specific mathematical models to reshape a time series. The second one is the set of qualitative transformations, whose aim is to quantize the dataset, without imposing particular models on the time series. The potential advantage of the quantization, which compresses a certain range of values of the data to a specific value, is that it helps avoiding the problems associated with the noise in the data, and it can make the analysis more robust to artifacts. However, the clear result is the loss of potentially valuable information.

The second category of variability techniques is what we called features. This term is commonly used in signal processing to identify a specific piece of information (feature) that can be extracted from the potentially available information in the signal. The aim of variability analysis is to extract a number of features from the data, and if needed (and possible given the amount and quality of the data) to track their temporal evolution. Features are not independent from transformations: in many cases, it is required to apply a transformation to the data before feature extraction, as this enables the extraction of features otherwise concealed or inaccessible. For example, the Fourier transform is a transformation, and from it one can extract multiple features like the total power of the signal, the power at specific frequencies and the power within specific frequency ranges.

The classification of techniques should ideally be oriented towards identifying families or groups of techniques which offer similar means of calculation and similar information content within groups, and distinct representations of the degree and character of the patterns of variation between groups. In addition, a classification system should be all-encompassing (able to incorporate all techniques), and physiologically relevant (offering assistance to understanding the physiology of healthy variability, and the pathophysiology of altered variability with illness). Distinct types of informative content have traditionally been labelled as domains of variability [13-17]. The most specific classification sees several domains, such as the time domain, the frequency domain, the entropy domain and the scale-invariant domain [13]. While this classification has proved useful, it does not encompass all the techniques of variability analysis available today. In this paper, we introduce a slightly broader classification in the hope of stimulating further discussion and research. Our review has identified five different domains of variability: **statistical**, for which the associated features describe the statistical properties of a distribution and which assumes the data are from a stochastic process; **geometric**, which describes those properties that are related to the shape of the dataset in a certain space; **energetic**, describing the features related to the energy or the power of the time-series; **informational**, describing the degree of irregularity/complexity inherent to the order of the elements in a time-series; **invariant**, describing the properties of a system that demonstrates fractality or other attributes that do not change over either time or space. The chart of the domains, together with the list of the features analyzed in this review, is reported in Table 1.

**Table 1:** Description of the domains characterizing the features

Types of domain	Name
Statistical	Standard statistical features, form factor, some symbolic dynamics features, turns count.
	
Geometric	Grid counting, heart rate turbulence, Poincaré plots features, recurrence plot features, spatial filling index.
	
Informational	Approximate entropy, conditional entropy, compression entropy, fuzzy entropy, Kullback-Leibler permutation entropy, multiscale entropy, predictive-based features, sample entropy, Shannon entropy, similarity indexes, Rényi entropy.
	
Energetic	Frequency features, energy operators, multiscale time irreversibility, time-frequency features.
	
Invariant	Allan scaling exponent, correlation dimension, detrended fluctuation analysis, diffusion entropy, embedding scaling exponent, Fano scaling exponent, finite growth rates, Higuchi's algorithm, index of variability, Kolmogorov-Sinai entropy, largest Lyapunov exponent, multifractal exponents, power spectrum scaling exponent, probability distribution scaling exponent, rescaled detrended range analysis, scaled windowed variance.

It is worthwhile to compare and contrast prior classifications with the proposed one. Time domain is divided into statistical and geometric, as was already proposed by the Task Force. The frequency domain is included together with features like the energy operators into the more general energetic domain; likewise, the entropy and the scale-invariant domains have become, respectively, part of the more general informational and invariant domains. Using terms and notions that are widely spread in the scientific community (e.g. information, invariance, energy), we hope this new classification enhances the previous classifications without introducing unnecessary complexity and/or confusion. For example, the predictive-based features find their place in the informative domain, instead of the more generic nonlinear domain. Similarly, the entropy features are inserted in the informative domain because they extract the same type of information, i.e. about the complexity of the system. Therefore, the number of domains remains practical, while improving the capability to classify new techniques through its generality. Moreover, despite the generality, the edges between the domains remain well-defined (e.g. the "time" domain of previous classifications was too generic). Adding the distinction between features and transformations, needed for clarification purposes, we believe that the proposed classification system represents a valid alternative to everything else currently available.

We will now analyze the different type of transformations and domains, describing the assumptions underlying each technique, discussing benefits and limitations.

## Transformations

### Quantitative

#### Power spectrum

The power spectrum is a method that assumes the signal under study comes from a stationary stochastic process. There are several methods to extract the power spectrum of a signal (i.e. frequency transformations). Among the ones used in clinical settings are the Blackman-Tuckey's method [22], the Welch's method [23], the Burg's method [24,25], Yule-Walker's method [26], the Lomb's method [27] and the multitaper transform [28,29]. The performance of these methods in the extraction of the power spectrum depends on the nature of the dataset under study, and its study represents a research field *per se *[30,31]. In general, it is not possible to select one technique to suit any particular application. Just to provide an example, above the ones cited, the Lomb method is the only one designed to create the power spectrum of an unevenly sampled time series; as such, it is preferable in the case of RR interval analysis, whenever interpolation should be avoided [27]. The above being classical signal processing techniques that have been extensively studied, their detailed analysis would be out of scope in this context; we suggest the interested reader to refer to [32] for a detailed explanation and comparison of their properties. Similar considerations can be made when higher order spectra (HOS) are studied. To explain in simple words what HOS are, it must be recalled that the power spectrum of a signal is equivalent to the Fourier transform of its autocorrelation function, i.e. the second order moment of the signal (via the Wiener-Khintchin theorem). Whenever the Fourier transform of moments higher than the second one are desired, a higher order spectrum can be obtained. HOS are particularly suited for the analysis of nonlinear systems and non-Gaussian signals [33,34].

#### Time-frequency

All the power spectrum estimation techniques presented so far are based on the assumption of absence of nonstationarities in the studied signals, implying that their properties do not change with time. In clinical settings this is not always the case, even if this rule is often neglected trying to study short-time recordings. To extend this analysis to nonstationary signals different techniques were introduced (i.e. time-frequency transformations). The most classical approach involves a windowed analysis of a time series, i.e. the division of the time series in multiple segments (also called windows). For every window the power spectrum is computed using the frequency transformations just cited. A standard is the short-time Fourier transform (STFT) [35], where the time-frequency transformation is computed just using the Fourier transform of the considered window. Because those methods relying on a windowed analysis suffer of the trade-off between frequential and temporal resolution (large windows give high frequential resolution but low temporal resolution, and vice versa), another class of techniques was introduced. The Wigner-Ville transform (WVT) [36] is a technique based on the Fourier transform of the instantaneous autocorrelation of the signal, and the wavelet transform (WT) [37,38] is based on the correlation at different scales and time between the data and a reference signal, called mother wavelet. The former is capable to give the finest temporal and frequential resolution compared to the other techniques, but is highly sensitive to noise; the second was developed to give high frequential resolution at low frequencies and high temporal resolution at high frequencies. Time-frequency transformations are signal processing techniques with a broad literature behind, therefore their detailed discussion is out of scope.

#### Integral pulse frequency modulation

The integral pulse frequency modulation (IPFM) is a model hypothesizing that the sympathetic and parasympathetic systems influences on the sino-atrial node can be associated to a single modulating signal, which integral generates a beat every time a threshold is surpassed [30]. Given a RR interval time series, from this model it is possible to extract the spectrum of the modulating signal, and from it multiple energetic features. The benefit of the IPFM model is a direct physiological explanation of the variability of a RR interval time series; the limitations consists in neglecting the effect of other systems (e.g. respiration, hormonal activity), and imposing a simple model describing the autonomic nervous system regulation of the heartbeat. The features that can be extracted from the IPFM model correspond to all the ones that will be presented for the power spectrum. This model was recently extended to enhance the computing of heart rate turbulence [39], a geometric feature successfully employed to assess the risk of myocardial infarction.

#### Phase space representation

The Phase space representation is a transformation mapping a time series into a multidimensional space, where each dimension represents an independent variable describing the system under study. There are several variants of this transformation [40], however the most famous is extracted from Takens' embedding theorem [41]. Taken's theorem justifies the transformation of a time series into a *m*-dimensional multivariate time series. This is done by associating to each *m *successive samples distant a certain number *τ *of samples, a point in the phase space. This technique is used for the characterization of dynamical systems, and it is mathematically proved that it can be used to recreate the attractor of the dynamics characterizing the signal (an attractor is a set of states in which the dynamics converge over time). The main limitation is that the attractor can be reconstructed on the assumptions that 1) the time series object of study is without noise and 2) infinitely sampled. Despite this is not the case for clinical applications, its usefulness is well assessed in the literature. The reason is that the phase space representation is mathematically equivalent to a multidimensional representation of the autocorrelation of the signal, making it useful also when the degree of determinism of a time series is not clear (e.g. heart rate variability [42]). This transformation is the starting point of other types of transformation, like recurrence plots, Poincaré plots, Grid transformation and certain types of Symbolic dynamics.

#### Recurrence plots

Recurrence plots are a visual representation of all the possible distances between the points in the phase space [43]. Whenever the distance between two points is below a certain threshold, there is a recurrence in the dynamics: i.e. the dynamical system visited multiple times a certain area of the phase space. From this transformation, well suited for the study of short nonstationary signals, many geometric features can be extracted.

#### Poincaré plots

The Poincaré plots are a particular case of phase space representation created selecting *m = 2 *and *τ = 1*; that corresponds to displaying a generic sample *n *of the time series as a function of the sample *n-1 *[9]. This is also known as a return map. The main limitation of this technique is that assumes that a low dimensional representation of a dynamical attractor is enough to detect relevant features of the dynamics. Despite its simplicity, this transformation was successfully employed also with high dimensional systems like the heart rate signals. The benefit is that, given the low dimensionality, it is possible to easily design and visualize several types of geometric features. A variant of the Poincaré plot is called the Angle map, which is a Poincaré plots made after that the transformation of the data into a polar coordinate system [44].

#### Grid transformation

The Grid transformation corresponds to the representation of the data into a bi-dimensional phase space (i.e. *m = 2*, with tunable embedding delay *τ*) that is divided into a fixed number of squares called pixels. This process creates a binary image of the phase space, where 0 is assigned to the pixels that were not visited by the dynamics, and 1 to the pixels that were visited [45].

#### Rhythmometric analysis

The rhythmometric analysis (RA) models the changes inside a time series as a set of cosine functions with linked frequencies [14,46]. There are three main components in this kind of model: circadian rhythm (with a given frequency *f*, considered as the fundamental frequency of the time series), infradian rhythms (which frequencies are below *f*) and ultradian rhythms (which frequencies are multiples of *f*). Therefore, RA is a frequential analysis that tries to explain the changes at different time scales of the data. The main difference with standard frequential analysis is that only particular frequencies are taken into account, and usually there are additional terms in the model that take into account the variability of the frequencies of the components inside the model. This transformation can be employed to explain the changes of features over time, but also to create a purely deterministic version of the original time series that can be studied more easily because of the absence of noise [14,46].

#### Point processes

Point processes are a family of stochastic models modeling the time intervals between successions of events. In this kind of model the information is held only in the intervals between the events, and not in the event *per se*. An RR interval time series by definition can be seen as a point process [47-49]. As any stochastic model, a certain probability distribution, in the case of point processes called Conditional Intensity Function, is used to draw at which time the next event (i.e. a beat) will take place. To follow the nonstationarity of the signal, the parameters of the model can be estimated through adaptive filtering [47-49].

### Qualitative

#### Bin transformation

In the case of a bin transformation, the range of the time series (determined by the minimal and maximal value of the variable) is divided in a specific number of equally spaced intervals of arbitrary size. Every time a data point falls in a given interval, the count in the associated bin is increased by one. In the case of power law analysis the time series are logarithmically rescaled before or after the bin transformation. This transformation is commonly employed in the construction of histograms.

#### Symbolic dynamics

The purpose of symbolic dynamics is to transform a time series into a sequence of specific symbols. Plenty of possible approaches can be used to coarse-grain a time series, however the basic idea is always to divide the range of the time series into intervals and assign to each interval a symbol. This process is usually employed either considering the signal as a whole or considering windows of the time series. When this process is separately applied to windows of the time series, the absolute information held by the signal is lost (i.e. detrend of the time series), keeping only the information related to its shape. Symbolic dynamics transformation is often applied in combination with the phase space representation; this approach corresponds to the division of the phase space into a set of hypercubes [25].

Being only a way to discretize the dataset, the symbolic dynamics transformation is associated to features belonging to different domains of variability. A set of features contains informational domain measures, usually applied also with the bin transformation (e.g. Shannon entropy, Rényi entropy, similarity based indexes). The other set contains statistical features that belong only to the symbolic dynamics. Each set of features will be discussed in the appropriate paragraph below.

## Features

### Statistical

The statistical domain assumes the data is from a stochastic process; the following features describe the properties of their distribution. For a summary of the key concepts, one can refer to Table 2.

**Table 2:** Summary of the statistical domain

Domain Assumptions	Features	Feature Assumptions	Transformation used	References
The information is held in the statistical descriptors of the data distribution (stochastic process)	Form factor	Gaussian distribution of the time series and its derivatives		[50,51]
	
	Symbolic dynamics features		Symbolic dynamics	[25,26,52-54,86]
	
	Standard statistical features	Gaussian distribution of the time series	Bins	[9,16-18,25,55-63]
	
	Turns count	Spike-like behaviour of the time series		[50]

### Form factor

This feature is based on the standard deviations of the signal and its first and second derivatives (a derivative represents an infinitesimal change in the signal). The computation involves the ratio between 1) the ratio of the standard deviations of the second and the first derivative of the signals, and 2) the ratio of the standard deviations of the first derivative and of the signal. The Form factor was found useful for the characterization of vibroarthrographic signals (sounds coming from the movement of the knee-joint, describing the mechanical properties of articular cartilage surfaces) [50] and the detection of arrhythmias [51].

### Symbolic dynamics features

Those are based on the transformation of a time series into a sequence of symbols through the association of a range of values with a specific symbol. There are two types of features characteristic of symbolic dynamics:

- Forbidden words, which counts the number of words (a succession of symbols) with a given length that do not appear. This technique was applied for the evaluation of heart rate and blood pressure variability in patients with dilated cardiomyopathy [52].

- Percentage of variations, which quantifies the number of variations inside the words composing a time series. For example, if words of 3 symbols are under study, the word [a a a] has no variations (all symbols are identical), the word [b b a] has one variation (two successive symbols are identical), the word [a b a] has two variations (there are no successive repetitions of symbols). This technique was applied in the characterization of autonomic cardiac modulation during arrhythmias [53] and the characterization of chronic heart failure [54].

Other features used with symbolic dynamics transformations can be found in the informational domain (such as Shannon entropy, see below).

### Standard statistical features

Those features were previously collected by the Task Force in 1996 to study heart rate variability [18]. They involve mean, standard deviation, counting of the samples above or below a certain threshold, and other statistical measures that are based on the distribution of the data and not their order (the list can be found in Table 3) [16,55-58].

**Table 3:** List of Standard statistical features

Short name	Description
SDNN	Standard deviation of the Normal-Normal (NN) interval.
	
RMSSD	Square root of the mean squared differences between each successive NN interval and the mean interval.
	
pNN x	The ratio between the number of successive NN intervals that are greater than × and the total number of NN intervals. Usually × is set either 20 ms or 50 ms [55].
	
NN x	It is the number of successive NN intervals that are greater than x. Usually × is set to 20 ms.
	
HTI	HRV Triangular Index: ratio of the total number of RR intervals to the number of intervals in the bin with the highest number of intervals The bin width should be around 1/128 seconds (128 Hz is the standard).
	
TINN	The triangular interpolation of the NN interval histogram (TINN) is the width of the base of the triangle that best approximates the NN interval distribution (the minimum square difference is used to find such a triangle).
	
IQRNN	Interquartile range of NN. It is the difference between the upper and the lower quartile values of the NN interval distribution (25% of the samples above and below the median value) [16].
	
SDSD	Standard deviation of the first derivative of the time series [56].
	
Conditional probability	Probability that given certain conditions, a specific event will occur [57,58]

To limit the complexity of the analysis, in the case of heart rate variability those measures are usually applied to the normal RR interval time series, where the prefix "normal" indicates the R peaks that are not classified as premature (i.e. ectopic beats) or extracted from known cardiac electrical dysfunctions (e.g. fibrillation). Despite their simplicity, the value of these measures has been proven in innumerable clinical applications like the prediction of sepsis [59], the assessment of the risk of myocardial infarction and diabetic neuropathies [18,60], the assessment of cardiac contractility [61], and the classification of cardiac death [25], chronic heart failure [17], severity of Parkinson's disease [9], asthma and chronic obstructive pulmonary disease [57,58,62,63].

### Turns count

Turns count records the number of times that the signal is above or below a certain threshold. This measure can be applied successfully only when specific properties of the signals are investigated. Following this reasoning, the threshold can be either fixed or adaptive. An adaptive threshold using the standard deviation of the analyzed signal was used to classify vibroarthrographic data from different knee-joint pathologies [50].

### Geometric

The geometric domain describes the properties that are related to the shape of the dataset in a certain space. For a summary of the key concepts refer to Table 4.

**Table 4:** Summary of the geometric domain

Domain Assumptions	Features	Feature Assumptions	Transformation used	References
The information is held in the specific position of the points represented in a reference space (deterministic system)	Grid counting	Low dimensionality of the attractor	Grid transformation	[45,64]
	
	Heart rate turbulence	Information is in the ectopic beats		[65]
	
	Poincaré plots features	Low dimensionality of the attractor	Poincaré plots	[4,9,54,61,66,67]
	
	Recurrence plot features	The attractor can be effectively described by the distances between its points	Recurrence plots	[19,43,68-70]
	
	Spatial filling index	The attractor is described by its degree of sparseness	Phase space representation	[71,72]

### Grid counting

This feature estimates how much a bi-dimensional attractor fills its phase space, and is based on the Grid transformation. Essentially, the Grid counting is the number of pixels visited by the dynamics divided by the number of pixels in the grid. The Grid counting was applied to the detection of ventricular fibrillation [45,64].

### Heart rate turbulence

Heart rate turbulence is a technique applicable only to RR interval time series. It is based on the evaluation of ventricular premature complexes (VPCs), and studies how VPCs modify the pattern of an RR interval time series. In normal subjects, the heart rate following a VPC incurs an early acceleration followed by a late deceleration. Heart rate turbulence extracts indexes describing those two phenomena through, respectively, the Turbulence onset and the Turbulence slope. The Turbulence onset is given by the percentage difference between the two RR intervals preceding and following a VPC, and is an index of early deceleration. The Turbulence slope is given by the maximum positive regression slope assessed over any 5 consecutive beats within the first 15 beats after the VPC, and is an index of late deceleration. The sequence of RR intervals before and after a VPC is called VPC tachogram. Given the variability between the heart rate pre- and post-VPCs, turbulence onset and turbulence slope are measured over the so-called average VPC tachogram, i.e. the average of the VPC tachograms over usually 24 hours (at least 5 VPCs are needed).

Heart rate turbulence was primarily applied to risk stratification after myocardial infarction and to the prediction of risk of heart failure. A list of other applications with future directions is provided in a recent review [65].

### Poincaré plot features

These features are based on an ellipse fitted to the Poincaré plot, which displays sample *n *of a time series as a function of sample *n-1*. These features can be seen as measures of nonlinear autocorrelation. If successive values in the time series are not linearly correlated, there will be a deviation from a line that is often properly modeled using an ellipse. The different features involve the centroid of the ellipse, the length of the two axes of the ellipse, the standard deviation in the direction of the identity line (called SD2), the standard deviation in the direction orthogonal to the identity line (called SD1) and their combination, namely cardiac sympathetic index (SD2/SD1) and cardiac vagal index (log_10_(16 × SD1 × SD2)). Those features have been successfully applied in the assessment of cardiac contractility [61], the classification of the severity of Parkinson's disease [9], the tracking of aging [4] and the assessment of chronic heart failure [54]. An additional feature that can be extracted from the Poincaré plot is the central tendency measure, which is the percentage of points which falls within a certain radius from the centre of the Poincaré plot of the first difference of the original time series. This feature was used to classify a variety of heart conditions and assess determinism in electroencephalographic signals [66,67].

### Recurrence plot features

A Recurrence plot is a visual representation of all the possible distances between the points constituting the phase space of a time series. There are four main elements characterizing a recurrence plot: isolated points (reflecting stochasticity in the signal), diagonal lines (index of determinism) and horizontal/vertical lines (reflecting local stationarity in the signal). The combination of these elements creates large-scale and small-scale patterns from which is possible to compute several features, mainly based on the count of number of points within each element. Table 5 provides a list of the main features used in clinical applications; for further features refer to Marwan et al. [43].

**Table 5:** List of Recurrence plot features

Short name	Description
%Recurrence	Represents the number of recurrences among the total number of possible recurrences.
	
%Determinism	Represents the number of recurrence points that are part of a diagonal of at least *l_min_*elements (usually *l_min _= 2*), divided by the number of recurrences.
	
%Laminarity	Corresponds to the computation of %Determinism, but considering vertical (or horizontal) lines instead of diagonal lines.
	
Trapping Time	Represents the number of recurrence points that are part of vertical (or horizontal) lines, divided by the number of vertical (or horizontal) lines.
	
%Determinism/%Recurrence	This ratio is often used to detect transitions of the dynamics.
	
Segment lengths	The mean and maximum lengths of diagonal and vertical (or horizontal) lines.
	
Shannon entropy	Same feature presented in the informational domain, but applied to the distributions of the lines (either diagonal or vertical/horizontal).
	
Kolmogorov entropy	The slope of the line fitting the log-log plot of the distribution of diagonal lines as a function of the threshold used to create the recurrence plot.

Recurrence plots have found application in the prediction of cardiac arrhythmia [19], event detection in EEG [43], diabetic autonomic dysfunction assessment [43,68], evaluation of the postural instability in patients affected by Parkinson [69], and are applicable to the assessment of many other physiological conditions [70].

### Spatial filling index

Based on the transformation of the time series in the phase space, this feature is extracted by first dividing this space into a set of identical hypercubes. The spatial filling index represents a measure of the number of points that on average are inside a hypercube, and describe how much the attractor fills the phase space. SFI was applied for the classification of normal heart rhythm, arrhythmia, supraventricular arrhythmia and congestive heart failure [71,72].

### Energetic

The energetic domain describes the features related to the energy or the power of the data. For a summary of the key concepts refer to Table 6.

**Table 6:** Summary of the energetic domain

Domain Assumptions	Features	Feature Assumptions	Transformation used	References
The information is held in the energy of the signal	Frequency features	Stationarity of the time series	Frequency transformations	[9,18,22,25,60,61,75]
	
	Time-frequency features		Time-frequency transformations	[9,18,22,25,60,61,75]
	
	Energy operators	The time series expresses periodicity		[73,74]
	
	Multiscale time irreversibility	The time series is created from a dissipative process, which dissipation can be quantified by the degree of temporal asymmetry		[76]

### Energy operators

These features combine the information of multiple points of the time series with nonlinear operations. Two features of this type are the Plotkin and Swamy operator, and Teager's operator. The latter is a specific case of the former; it is more sensitive to noise but mathematically linked to the energy of a sine wave, defined as a function of the product of its amplitude and its frequency. This fact motivates the name of these features. Despite the fact that these features were developed for sinusoidal signals, in clinical settings their application has been extended to signals showing periodicities: the Plotkin and Swamy operator was applied to segmentation and feature extraction of EEG signals [73], and the Teager's operator to the detection of peaks for the estimation of foetal heart rate from phonocardiographic signals [74].

### Frequency and time-frequency features

The frequency features are the ones that can be extracted from any frequency transformation. The standard approach is to divide the spectrum in different bands (frequency ranges), and calculate the central frequency in each range, i.e. the peak in the power spectrum [18]. Beside the maximum value, other statistics include the integral over specific bands as well as different types of ratios between the cited features. For example, in the case of RR interval time series, the power spectrum is usually divided into four bands with the following frequency ranges: ultra low frequencies < 0.003 Hz, very low frequency 0.003-0.04 Hz, low frequencies 0.04-0.15 Hz, and high frequencies 0.15-0.4 Hz. Some works do not follow the guidelines, preferring different bands [75]. Common features involve the power in the low and high frequency ranges, and their ratio [18,60]. The time-frequency transformations represent the extension of the frequency transformations to the class of nonstationary signals. Because they also describe the energy of the signal at certain frequencies, the time-frequency features are identical to the frequency features.

Those types of features are among the most used, and their applications involve among others the assessment of the risk of myocardial infarction and diabetic neuropathies [18,60], the characterization of heart rate variability during haemodyalisis [22], the assessment of cardiac contractility [61], the classification of cardiac death [25] and the severity of Parkinson's disease [9].

### Multiscale time irreversibility

Multiscale time irreversibility quantifies the degree of temporal asymmetry of a signal, i.e. how much energy is dissipated during the development of the process. In time series analysis, this corresponds to the modification of the statistical properties of a signal under the operation of time reversal. Multiscale time irreversibility is based on the computation of several differential time series derived from the original one. That means taking a sample at time *i+j *and subtracting from it the value of the sample at time *i*, then repeating this for all the samples *i*; the process has to be repeated with different values of *j *(representing the scale parameter), creating a set of time series. The percentage of difference between the increments and decrements inside each time series is computed; the sum of all these percentages gives the multiscale time irreversibility [76]. Even if created for the study of heart rate variability, this measure has not been fully characterized in specific clinical settings.

### Informational

The informational domain describes the degree of irregularity/complexity inherent to the order of the elements in a time-series. For a summary of the key concepts, refer to Table 7.

**Table 7:** Summary of the informational domain

Domain Assumptions	Features	Feature Assumptions	Transformation used	References
The information is held in the degree of complexity, therefore: distance from periodicity and stochasticity, distance from a reference model, distance from a precedent pattern within the data	Approximate entropy			[4,71,77-79]
	
	Conditional entropy		Bins	[25]
	
	Compression entropy			[17,81]
	
	Fuzzy entropy			[77,80]
	
	Kullback-Leibler permutation entropy		Symbolic dynamics and phase space representation	[82]
	
	Multiscale entropy	The complexity changes depending on the window length used in the analysis		[76,83]
	
	Predictive-based features	The data follows a model, and the deviation (prediction error) from that model describes changes in the system.	Multiple	[56,84,85]
	
	Sample entropy			[4,71,77-79]
	
	Shannon entropy		Bins	[25,52,53,56,86]
	
	Similarity indexes	The comparison of the properties of two successive windows allows the detection of changes in a time series	Multiple	[26,45,87,88]
	
	Rényi entropy	Bins	[25]	

### Approximate entropy, Sample entropy and Fuzzy entropy

Approximate entropy (AP) is the negative natural logarithm of the conditional probability that a dataset of length N, having repeated itself for *m *samples within a tolerance *r*, will repeat itself again for one extra sample. A window of length *m *is run along the signal to generate a set of data vectors of length *m*. One then computes the number of times that the Euclidean distance between all pairs of these vectors is less than a threshold *r*. This is repeated for windows of length *m+1*, and the logarithm of the ratio of these two numbers is taken. AP is the precursor of sample entropy (SE); the difference is that SE excludes the counts where a vector is compared with itself. This avoids the bias these self-matches introduce in the estimation. Given this improvement, SE should be always preferred to AP. To avoid the sensitivity to the threshold *r*, a new entropy called fuzzy entropy (FE) was developed. All the computational steps are the same, with the difference that SE uses *r *to produce a binary classification of the distance between two windows (zero if they are more distant than *r*, one otherwise), while FE uses a fuzzy membership function to evaluate the distance. This continuous function scores as one if the distance between two windows is infinitesimal and decays exponentially to zero the more distant are the vectors. This improvement avoids the discontinuity of the binary classification, therefore lowering the sensitivity to the threshold. Chen et al. analyzed the three entropies on synthetic datasets, demonstrating the improved performances of FE on the characterization of synthetic datasets with different mixtures of determinism and noise [77].

AP and SE have been applied to several fields like the analysis of the EEG [78], the fluctuations of the center of pressure in static posturography [79], the characterization of heart rate variability in patients undergoing sepsis shock [4] and for the classification of multiple heart disorders [71]. On the contrary, fuzzy entropy has been applied mainly in the characterization of EMG signals [80].

### Conditional entropy

Consider two random variables, say *X *and *Y*. The conditional entropy of *Y *given *X *is defined as the difference between the joint entropy of *X *and *Y *(that is the entropy of the union of *X *and *Y*), and the entropy of *X*. To provide an example, consider a time series reconstructed in a *m*-dimensional phase space. *X *can be the set of points in the *m*-dimensional space, and the union of *X *and *Y *can be the set of points given by the reconstruction of the time series in a *m+1*-dimensional phase space. The entropy used for the calculation of the conditional entropy can be any one reported in this paper. In [25] the conditional entropy was computed using the Shannon and the Rényi entropies for the classification of cardiac death.

### Compression entropy

Compression entropy is a readjustment of a compression algorithm called LZ77. After the transformation into symbols, the sequence is compressed and the entropy is computed as a function of the ratio between the length of the compressed signal and the length of the original signal. This technique was used for the classification of ventricular tachycardia [81] and chronic heart failure [17].

### Kullback-Leibler permutation entropy

The permutation entropy is based on the representation of the time series in a symbolic phase space. Considering a single point in the phase space, the idea is to substitute to each coordinate the rank among the coordinates. For example, the vector [0.3 0.2 0.7] would be substituted to the vector [2 1 3], because 0.2 is the smallest element of [0.3 0.2 0.7], therefore it is exchanged with a 1; its largest element 0.7 is exchanged with a 3; and 0.3 is the element in between, exchanged with a 2. The process is repeated for each point in the phase space, creating a set of words. Then, the Shannon entropy of the set of words is computed, creating the so-called permutation entropy. A normalization using the total number of possible words achievable in that phase space representation, leads to the Kullback-Leibler permutation entropy. This technique has been applied to the characterization of foetal heart rate variability [82].

### Multiscale entropy

As defined in [76], "multiscale entropy quantifies the information content of a signal over multiple time scales". Consider a non-overlapping window analysis of the original time series, where the sample mean inside each window is computed. This set of sample means constitutes a new time series. Repeating the process *N *times with a set of window lengths starting from 1 to a certain length *N*, this will give a set of *N *time series of sample means. The multiscale entropy is obtained by computing any entropy measure (sample entropy is suggested) for each time series, and displaying it as a function of the number of data points *N *inside the window (i.e. of the scale). The authors suggested focusing on the analysis of the resulting curves rather than on extracting a single parameter. However, to assist clinical classification in the case of RR interval time series, they proposed to extract the slopes of the curve over the scales from *N = 1 *to 5, and from *N = 6 *to 20 [83]. Multiscale entropy was also applied to the comparison of sleep-wake cycles of patients with heart failure, young and elderly [83].

### Predictive-based features

A predictive-based feature is any error that a model produces when trying to fit or predict the data. Models are based on hypotheses; therefore, in the case where the distance between the signal produced by the model and the real signal (i.e. the error) is high, the time series is less compatible with the underlying hypotheses of the model. The error can be evaluated through specific cost functions (e.g. the mean squared prediction error, prediction gain, sum of the residuals) and can be referred as an index of complexity [84]. Because the modeling of physiological signals is entirely another field of research, we will not address this topic further. Examples of this approach applied to RR interval time series are the usage of the residuals of an autoregressive model [56], as well as of the prediction error from local nonlinear predictors [84] and predictors based on conditional distributions [85].

### Shannon entropy and Rényi entropy

As classical measures taken from information theory, these entropies are estimated after the transformation of the dataset into bins or a symbolic sequence. Counting the relative frequency of each bin/word, the Shannon entropy is estimated as the sum of the relative frequencies weighted by the logarithm of the inverse of the relative frequencies (i.e. when the frequency is low, the weight is high, and vice versa). A generalized version of the Shannon entropy is the Rényi entropy, which corresponds to the sum of the inverse of the relative frequencies elevated to a certain power *q ≥ 2*.

In the case of the bin transformation, Shannon entropy was applied in the classification of the heart rate variability of young/elderly and patients suffering from coronary artery disease [56]. For the symbolic transformation, Shannon entropy was applied to the detection of ventricular tachycardia or fibrillation [86], the characterization of autonomic cardiac modulation during arrhythmias [53], and the evaluation of heart rate and blood pressure variability in patients with dilated cardiomyopathy [52].

### Similarity indexes

The similarity indexes are features quantifying the "distance" (i.e. difference) between two time series, according to different metrics. The two time series can be either two different time series, or two segments from one time series. Usually the distance is calculated after a qualitative transformation of the dataset, and is expressed as a combination of the probability distributions of the words/bins or a combination of the entropy of the words/bins. When a windowed analysis of a single time series is pursued, the idea is to compare either the similarity between two successive windows, or the similarity between a window for a given physiological state (e.g. normal) and all the other windows, in order to track how the system is changing (e.g. moving from physiological to pathological). Examples of similarity indexes with associated clinical applications follow: 1) A similarity index defined as the sum of the product of the bins within the distributions of each segment was used in the assessment of anesthetic depth [87]; 2) A similarity index defined as the difference between the Shannon entropies of the symbolic distributions of each segment was used in the assessment of the effect of aging, atrial fibrillation and heart failure in heart rate variability [88], and the detection of arrhythmias [26]; 3) Considering two segments after a grid transformation as two images, it is possible to define similarity indexes based on image processing. Among the several proposed, one that worked effectively in the detection of ventricular fibrillation was the count of the pixels that were visited in both the considered images [45].

### Invariant

The invariant domain describes the properties of a system that demonstrates fractality or other attributes that do not change over either time or space. For a summary of the key concepts refer to Table 8.

**Table 8:** Summary of the invariant domain

Domain Assumptions	Features	Feature Assumptions	Transformation used	References
The information is held in those properties of the time series that are invariant-i.e. not supposed to change over either time or space.	Allan scaling exponent	The time series is modeled as a point process, and the ratio between the second order moment of the difference between the number of events of two successive windows and the mean number of events has scale-invariant properties	Point process	[49,89,90]
	
	Detrended fluctuation analysis	The standard deviation of the detrended cumulative time series has scale-invariant properties		[4,63,92-94]
	
	Diffusion Entropy	The time series is modeled as a family of diffusion processes, which Shannon entropies has scale-invariant properties		[54,95-97]
	
	Embedding scaling exponent	The variance of the attractor at different embedding dimensions has scale-invariant properties	Phase space representation	[98]
	
	Fano scaling exponent	The time series is modeled as a point process, and the variance of the number of events divided by the mean number of events has scale-invariant properties	Point process	[49,89,90]
	
	Higuchi's algorithm	The length of the time series at different windows has scale-invariant properties		[54,101-104]
	
	Index of variability	The time series is modeled as a point process, and the variance of the number of events has scale-invariant properties	Point process	[2]
	
	Multifractal exponents	Multiple scaling exponents characterize the time series	Wavelet transform	[54,105,106]
	
	Power spectrum scaling exponent	Stationarity, the power spectrum follows a 1/f^b ^like behaviour	Power spectrum	[92]
	
	Probability distribution scaling exponent	The distribution of the data has scale--invariant properties	Bin transformation	[63,107]
	
	Rescaled detrended range analysis	The range (difference between maximum and minimum value) of a time series has scale-invariant properties		[92]
	
	Scaled windowed variance	The standard deviation of the detrended time series has scale-invariant properties		[92]
	
	Correlation dimension	The time series is extracted from a dynamical system, and the number of points in the phase space that are closer than a certain threshold has scale-invariant properties	Phase space representation	[40,91]
	
	Finite growth rates	The time series is extracted from a dynamical system, which is described by its dependence on the initial conditions (how two points that are close in space and time separate after a certain amount of time)-the ratio between the final and the initial time is an invariant of the system	Phase space representation	[86]
	
	Kolmogorov-Sinai entropy	The time series is extracted from a dynamical system, and it is possible to predict which part of the phase space the dynamics will visit at a time t+1, given the trajectories up to time t	Phase space representation	[40,91]
	
	Largest Lyapunov exponent	The time series is extracted from a dynamical system, which is described by its dependence on the initial conditions (how two points that are close in space and time separate after a certain amount of time)-the distance grows on average exponentially in time, and the exponent is an invariant of the system	Phase space representation	[40,41,99,100]

### Allan and Fano scaling exponents

Those two features were created to characterize the fractal properties of a point process. The idea is to count the number of events in non-overlapping windows of the signal of fixed time-length *T*. The Fano factor at time *T *is the variance of the number of events divided by the mean number of events (it also called index of dispersion). The Allan factor at time *T *is the ratio between the second order moment of the count differences between two successive windows, and the mean number of events. The slope of the line fitting the log-log plot of the factor as a function of *T *gives, respectively, the Fano and the Allan scaling exponents [49,89]. These parameters were applied to the characterization of supraventricular arrhythmia and congestive heart failure [90].

### Correlation dimension

The correlation sum of a time series is defined as the number of points in the phase space that are closer than a certain threshold *r *[40]. An array of correlation sum values is then created by repeating the process for a certain range of thresholds. One then computes the correlation dimension as the slope of the line fitting the log-log plot of the correlation sum as a function of the threshold. This feature gives a measure of the dimensionality of the attractor, and was used to study blood pressure and mean cerebral blood flow velocity in diabetic autonomic neuropathy [91].

### Detrended fluctuation analysis

DFA is a technique quantifying how the fluctuations of a signal scale with the number of samples of that signal. First, the cumulative version of the time series is created, where the value of the original time series at a given point is replaced by the sum of values up to and including that point. The idea is to divide this cumulative time series into all the possible non-overlapping windows of length *m*, then detrend each window and compute the standard deviation within each window (i.e. the magnitude of fluctuations). Each window is detrended by removing the regression line fitting the data. The average of the standard deviation computed in each window is stored. Repeating the process for a certain set of window lengths yields a time series that may exhibit scaling behaviour, i.e. where fluctuations scale according as a power of the window length [92]. Several features can be extracted from DFA: (1) the slope of a line fitting the log-log plot of the standard deviation as a function of the window length, which expresses the so-called Hurst exponent [92]; (2) the local derivative in each point of the log-log plot [93].

For the heart rate variability (HRV) additional features arise from the division of the log-log plot in two areas, to extract two scaling exponents. Indeed, in different studies, two scaling exponents more properly fitted the DFA of the HRV than the standard slope of the log-log plot. Another particular application of DFA to heart rate variability uses, instead of the raw data, the magnitude and sign of the derivative of an RR interval time series [94].

DFA is one of the most used techniques in variability analysis, and was applied to the evaluation of posture, exercise and aging [93], sleep stage classification [94], the prediction of sepsis [4], and classification of asthma and chronic obstructive pulmonary disease [63].

### Diffusion entropy

Assuming the time series is a diffusion-type stochastic process, this measure quantifies its scaling behaviour over time. The first step is to calculate the diffusion trajectories, which are particular types of cumulative time series extracted from the original. Then, the probability distribution at a given cumulative time is built from the values of all the diffusion trajectories at that time, and the Shannon entropy is computed at that time. Repeating the procedure for all the times, a set of Shannon entropies as a function of time is created. The slope and offset of the line fitting the normal-log plot of the Shannon entropies as a function of the cumulative time gives information regarding the scaling behaviour of the system [95]. This technique was applied to the characterization of congestive heart failure [54,96,97].

### Embedding scaling exponent

This technique estimates how the variance of the time series reconstructed in the phase space changes with the value of the embedding dimension. The variance is computed from the diagonal of the covariance matrix of the time series reconstructed in the phase space. The slope of the line fitting the log-log plot of the variance as a function of the embedding dimension gives the embedding scaling exponent. The ESE has been applied to gait analysis of patients with Huntington's disease [98].

### Finite growth rates and largest Lyapunov exponent

After the representation of the time series in the phase space, the neighbourhood of each point in the phase space is computed. The idea is to compute how the considered point and its neighbour separate after a certain time *t*; the separation is measured using the Euclidean distance between the points. The measure used by the finite growth rates is the ratio between the final (i.e. after *t*) and the initial distance of the two points. The repetition of this process to all the points in the phase space gives a set of ratios, whose average corresponds to the finite growth rates (FGR). This technique was used for the detection of ventricular tachycardia or fibrillation [86].

Consider now that the finite growth rate is computed without the normalization by the initial distance (therefore, the finite growth rates now represent a final distance). If all the FGR for any time *t *are computed, the slope of the line fitting the log-log plot of the FGR as a function of *t *gives the largest Lyapunov exponent. The largest Lyapunov exponent expresses the exponential rate of divergence of the trajectories in the system, starting with two infinitely close points in the phase space [40,41]. Theoretically, the largest Lyapunov exponent is positive only in the case of chaotic systems, but this result is not assured when considering noisy time series. This dynamical-invariant feature was used for gait analysis [99], and congestive heart failure and atrial fibrillation evaluation [100].

### Higuchi's algorithm

The time series is divided into non-overlapping segments of *m *samples. Consider the samples in the *i*-th position inside each segment. The distance between those samples can be calculated as their absolute difference. If the normalized sum of all the distances of the samples in the *i*-th position is calculated for each position, and then averaged, the result is a measure of the length of the time series. The Higuchi's algorithm involves computing the length of the time series over a certain range *m*. The slope of the line fitting the log-log plot of the lengths as a function of *1/m *gives a measure of the fractal dimension of the time series, i.e. how the length of the time series scales. In the literature many other algorithms are available for the estimation of the fractal dimension of a time series, although Higuchi's is one of the most used in the biomedical domain [101-103]. This technique was applied to detect events in EEG [104], and characterize heart rate variability in chronic heart failure patients [54].

### Index of variability

The index of variability is based on a point process model. The time series is divided in non-overlapping windows of a given length, and for each window the number of events is counted. Then, the variance of the number of events for that window length is computed. The procedure is repeated for different window lengths. The scaling behaviour is extracted from the derivative of the variance of the number of events as a function of time. Index of variability was applied to evaluate the scaling properties of traffic processes in network engineering [2].

### Kolmogorov-Sinai entropy

The Kolmogorov-Sinai entropy is a measure expressing the information needed to predict which part of the phase space the dynamics will visit at a time *t+1*, given the trajectories up to time *t *[40]. Consider the natural logarithm of the ratio of the correlation sum computed for an embedding dimension *m*, with the correlation sum computed for an embedding dimension *m+1*. The plateau of the curve of this ratio as a function of the logarithm of the threshold gives the KS entropy. This measure was used to study blood pressure and mean cerebral blood flow velocity in diabetic autonomic neuropathy [91].

### Multifractal exponents

Multifractal time series are characterized by a set of different local scaling exponents (i.e. different scaling exponents at different times). One of the most used techniques to evaluate multifractality is the wavelet transform [105]. The idea is to generate the wavelet transform by using a particular type of mother wavelet, called wavelet leader, and from it extract the multifractal spectrum. This method was applied to the characterization of heart failure [54,105], and intrapartum diagnosis of fetal asphyxia [106].

### Power spectrum scale exponent

This feature represents a classical way to compute the Hurst exponent of a time series, and is computed as the slope of the line fitting the log-log plot of the power spectrum of the time series as a function of the frequency. This gives a reliable estimate only when the underlying process belongs to the fractional Gaussian noise family, and only for a specific range of the Hurst exponents; otherwise, the use of this feature should be avoided [92]. Because this technique extracts the Hurst exponent, as DFA does, they share the same applications. The same applies to the other two measures that were found more useful in the extraction of the Hurst exponent [92], i.e. scaled windowed variance and rescaled detrended range analysis. The difference between the four techniques is given only by the reliability of the estimate, which depends on the type of signal under study.

### Probability distribution scaling exponent

The shape of the probability distribution of a time series can be estimated taking the histogram of the time series, and normalizing it to a unitary area. When the representation in a log-log plot of the probability distribution shows a linear trend, it is possible to extract a scaling exponent by fitting a line to the distribution and taking its slope. In such a case, the tail of the distribution is larger than it would be if the time series had a Gaussian distribution, making rare events more likely to occur. This technique was applied to detect maturational changes in the respiration of infants [107]. Similarly, the scaling exponent can be extracted by fitting a probability density function with scale-invariant properties (e.g. Pareto or Levy distribution). The latter approach was used to discriminate between patterns of respiratory impedance in patients with asthma, chronic obstructive pulmonary disease and controls [63].

### Scaled windowed variance

Scaled windowed variance involves the same mathematical steps as DFA, with the difference that the original times series is studied, rather than its cumulatively integrated version. Despite the similarity, scaled windowed variance is preferable to DFA when the underlying process belongs to the fractional Brownian motion family [92].

### Rescaled detrended range analysis

Rescaled detrended range analysis is similar to DFA. Instead of calculating the standard deviation on each window, rescaled detrended range analysis computes the "range", defined as the difference between the maximum and the minimum values of the samples in the window. RDRA is preferable to DFA when the underlying process belongs to the family of persistent fractional Gaussian noise [92].

## Challenges

The wide array of techniques discussed in our review represents a starting point for advanced variability analyses. Clearly, more investigations are required to untangle all the information hidden within biological signal variability. Two grand challenges stand out, which we believe represent important future goals of variability analysis:

### 1) Reduction of the dimensionality of variability analysis for a practical use in clinical settings

Biological variability and variable physiological conditions motivate the use of different descriptors, capable of describing in the most accurate way possible the system under study. However, the collection of this huge array of univariate and multivariate techniques calls for the need to personalize the relevant information for each single subject, considering specific stress conditions that could be either pathological or not (e.g. physical exercise). A step toward this result would be given by the characterization of the nature and degree of interdependence between measures of variability in specific clinical situations. Few studies have tried to face this challenge in a broad way. The reason is that it is still a common practice to use a selection of few variability measures in order to reduce the complexity of the study. Indeed, the link between physiology and variability still needs to be understood more deeply, with the consequence that several researchers prefer to focus their analysis on small set of techniques. An example, such a characterization has been provided by Maestri et al. (2007), who performed a correlation analysis including only a small set of nonlinear, univariate variability techniques applied to the assessment of heart failure [54]. We believe that a more extensive characterization would enable the selection of the optimal set of descriptors for a given clinical situation, thereby reducing the complexity of the studies while still preserving all the valuable information. These would form the basis for advanced types of data reduction and fusion techniques, with the final aim to converge the different pieces of valuable information in an easy-to-use tool for physicians and nurses. While this information gets created through research, another approach could be the introduction of pattern recognition and data reduction methods, with the aim of creating tools capable of selecting features and thereby extracting information of specific clinical interest. The complexity of this task is even higher because almost all those techniques require additional choices to properly work (e.g. the principal component analysis requires a criterion to select the number of components to keep; neural networks require the definition of the number of neurons to be used, as well as their activation function). Therefore, a further challenge would be to make those tools capable of operating effectively in an automatic or semi-automatic way, further simplifying the clinical practice. The design of user friendly algorithms remains a main challenge, and we hope that our review can help with such developments along the lines of e.g. Jovic et al. [108].

### 2) Extension of variability analysis to multivariate techniques characterizing multi-organ variability and connectivity

In the scientific community it is well accepted that the human body is a complex system, and its behavior depends on the interactions of its components [13,109-111]. In spite of this fact, the common approach is to use univariate techniques [112,113], and this review is a proof of it. Examples of multivariate studies exist, however they are mainly focused on the evaluation of interactions between at maximum two systems, like the cardiac and respiratory systems through frequency analysis (spectral, wavelet...) [114-116], or the evaluation of the differences between heart rate variability and blood pressure [117], pulse oximeter data [22] and brain activity recorded through electroencephalography [118]. Multiorgan variability and connectivity, respectively defined as the ensemble of patterns of variation over time and interconnection over space, represent new tools to describe the behavior of the human body as a whole [119]. These tools will likely provide complementary information to that given by standard univariate techniques, and the simultaneous use of the two approaches together holds the promise of new clinically valuable results.

## Conclusion

Physiological time series are complex entities characterized by a variety of properties, some of which are often intertwined, such as through a combination of stochasticity and determinism. This complexity demands the use of a vast array of techniques, capable of describing in the most accurate way possible the time series under study. In this article more than 70 variability techniques were presented, explaining with an intuitive approach the idea behind them, discussing their limitations and associating references related to their clinical applications. Moreover, a novel way of classifying these variability techniques was proposed, specifically according to whether they perform transformations of the data or extract features from the data. The paper further highlights two grand challenges that the scientific community will face in the context of variability analysis for clinical applications.

## Competing interests

AJE Seely founded Therapeutic Monitoring Systems in order to commercialize patented Continuous Individualized Multiorgan Variability Analysis (CIMVA) technology, with the objective of delivering variability-directed clinical decision support to improve quality and efficiency of care.

## Authors' contributions

AB analysed current literature and prepared the manuscript. AB proposed the classification of the variability techniques, with the supervision of AJES and AL. AJES and AL supervised, revised and gave the final approval of the manuscript. All authors read and approved the final manuscript.
